# Supplementary material for: Anterior cingulate and medial prefrontal cortex oscillations underlie learning alterations in trait anxiety in humans
Source: Commun Biol. 2023 Mar 15;6:271. doi: 10.1038/s42003-023-04628-1 (PMC10017780; doi:10.1038/s42003-023-04628-1)
Supplement: Supplementary file 1 — Supplemental Material [file 42003_2023_4628_MOESM1_ESM.pdf]

Supplementary Figures

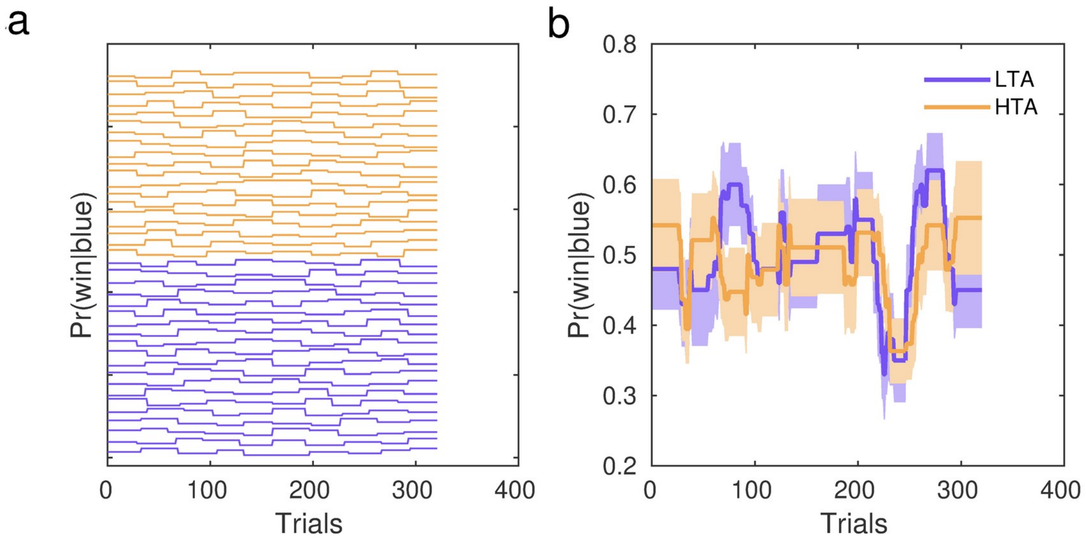

Supplementary Figure 1. a) Individual time courses of stimulus-outcome probabilistic relationships for individuals in the high trait anxiety (HTA, orange) and low trait anxiety (LTA, purple) groups. **b)** The probability governing the likelihood of the blue stimulus being rewarded, Pr(win|blue), across 320 trials is displayed as mean (and SEM) within each group. The mean traces in (b) represent the group-average of the individual traces in (a). There were no systematic (or significant) differences between HTA and LTA in the contingency mapping values ($P > 0.05$). Furthermore, Bayesian statistical analysis provided strong evidence that the two population means in this quantity were equal (**Supplementary Results**).

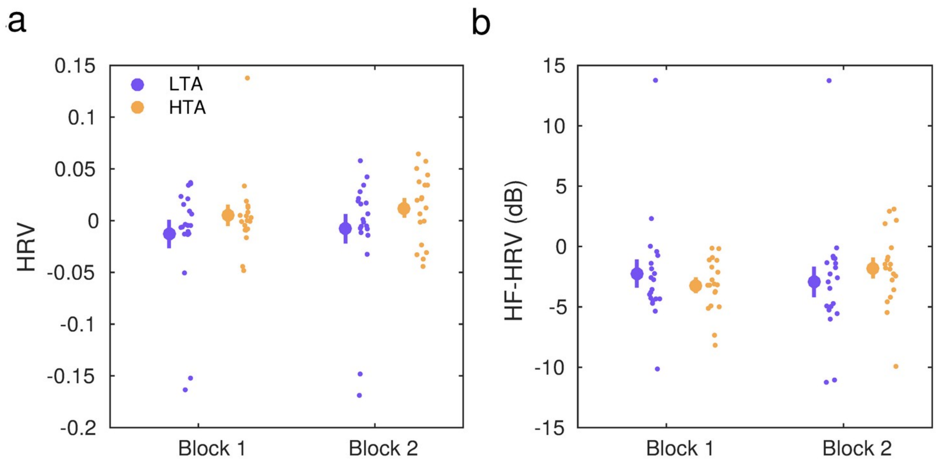

Supplementary Figure 2. Heart-rate variability in trait anxiety. a) Normalised heart-rate variability (HRV). Average HRV (coefficient of variation of the inter-beat-interval of the R-peak in the ECG signal) in high trait anxiety (HTA, yellow) and low trait anxiety (LTA, dark blue). The HRV from both experimental task blocks has been normalised by subtracting the average HRV in the resting state baseline (R1). No significant differences were found using a non-parametric 2 x 2 Block x Group factorial analysis with synchronised rearrangements ($P > 0.05$ for main and interaction effects). **b)** Normalised high-frequency (HF) HRV. Analysis of the high frequency (0.15–0.40 Hz) spectral content of the inter-beat-

interval (IBI) time series data revealed there was no significant difference between HTA relative to LTA ($P > 0.05$ as in a).

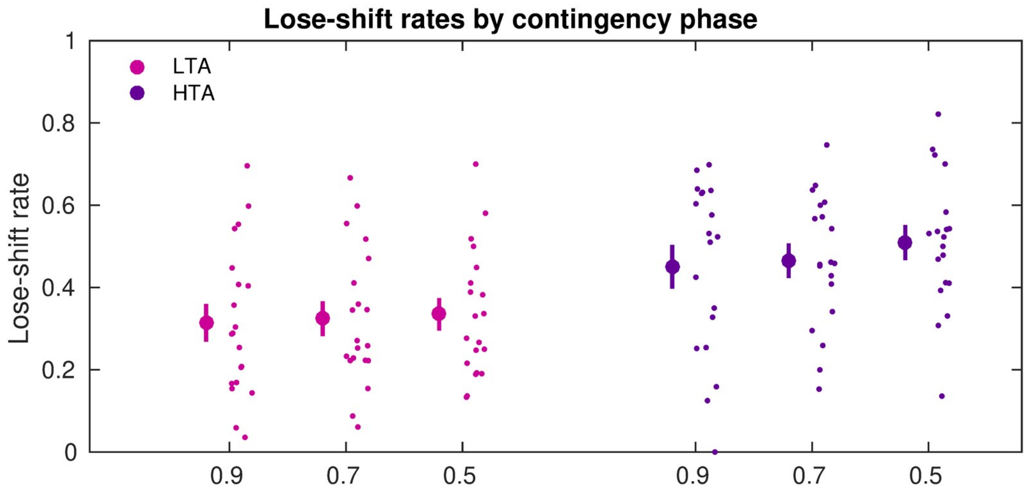

Supplementary Figure 3. Lose-shift rate across contingency phases. Illustration of the lose-shift rate, as in Figure 1D, across contingency phases: 0.9/0.1 and 0.1/0.9, 0.7/0.3 and 0.3/0.7, 0.5/0.5. Both groups (LTA: magenta; HTA: purple) of participants exhibited similar lose-shift rates across changes in stimulus-reward mappings.

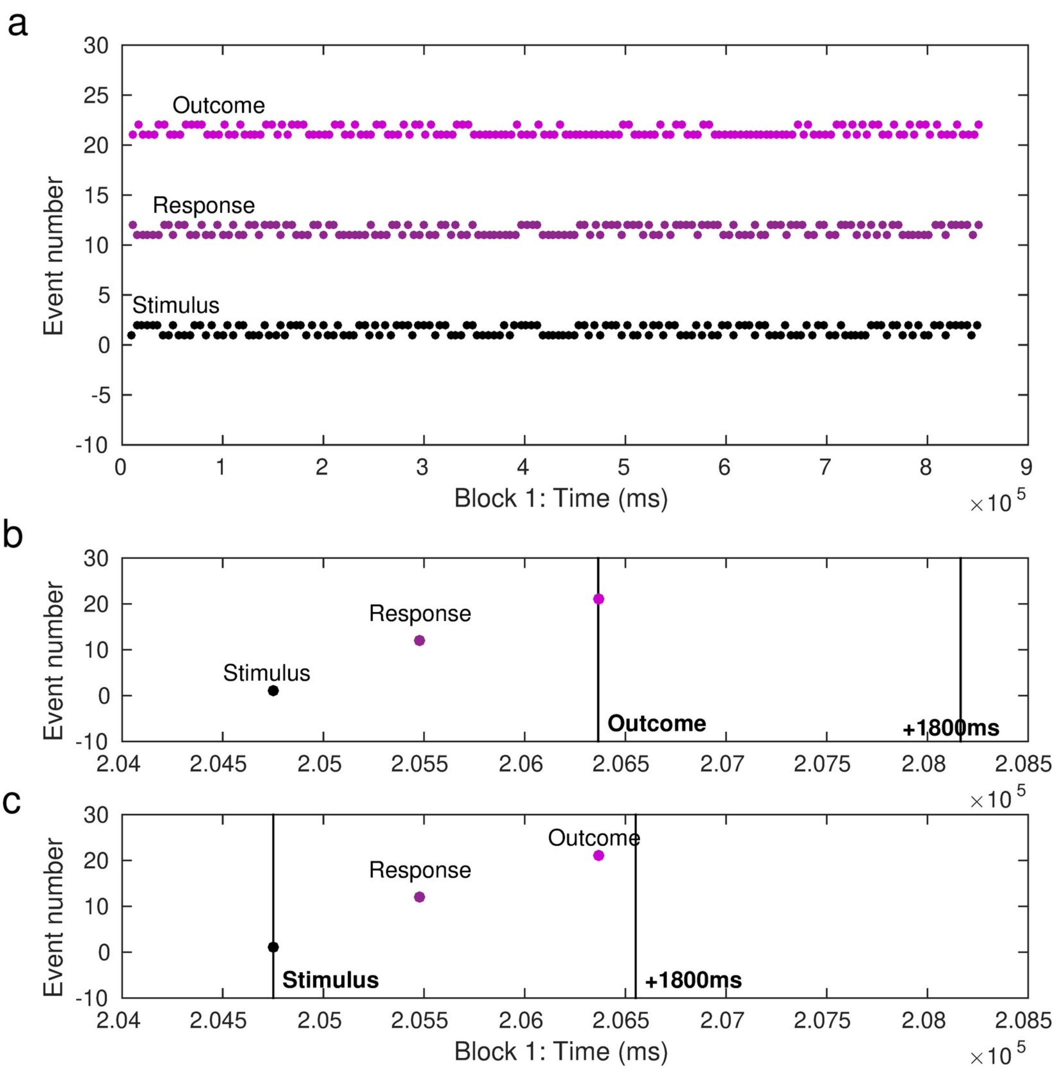

Supplementary Figure 4. Scheme of the timeline of expected effects of parametric and discrete regressors. a) Time course of events during completion of block 1 in a representative participant. The event (trigger) numbers are presented for stimuli (blue fractal on the right or left side; denoted by blue dots), responses (left, right, no response; dark purple), and outcomes (win, lose, no response; denoted by lighter purple). **b)** We hypothesised that the effects of the parametric regressors $|\varepsilon_2|$, σ_2, and σ_3, would be observed in an outcome-locked interval, within 0.2–1.8 seconds, based on ref.[34]. We estimated the GLM from -0.5 to 1.8 s around the outcome events and focused the statistical analysis on the interval 0.2–1.8 s. The resulting TF images were baseline corrected using as baseline level the activity from -300 to -50 ms. The vertical lines denote the outcome onset and the end of our interval of analysis at 1.8 s. **c)** In a separate exploratory analysis of the effect of predictions on the time-frequency responses, we used a stimulus-locked GLM with parametric regressor $|\hat{\mu}_2|$. We had hypothesised that the neural oscillatory processes correlated with predictions about the tendency of the stimulus-outcome contingency would be observed after the stimulus presentation, and before the outcome presentation. This GLM was conducted up to 1.8 s (vertical line) from the stimulus onset, but the statistical analysis focused on the range 0.2 to 0.7 s, based on our previous work[34]. TF images were normalised with the pre-stimulus interval -300 to -50 ms.

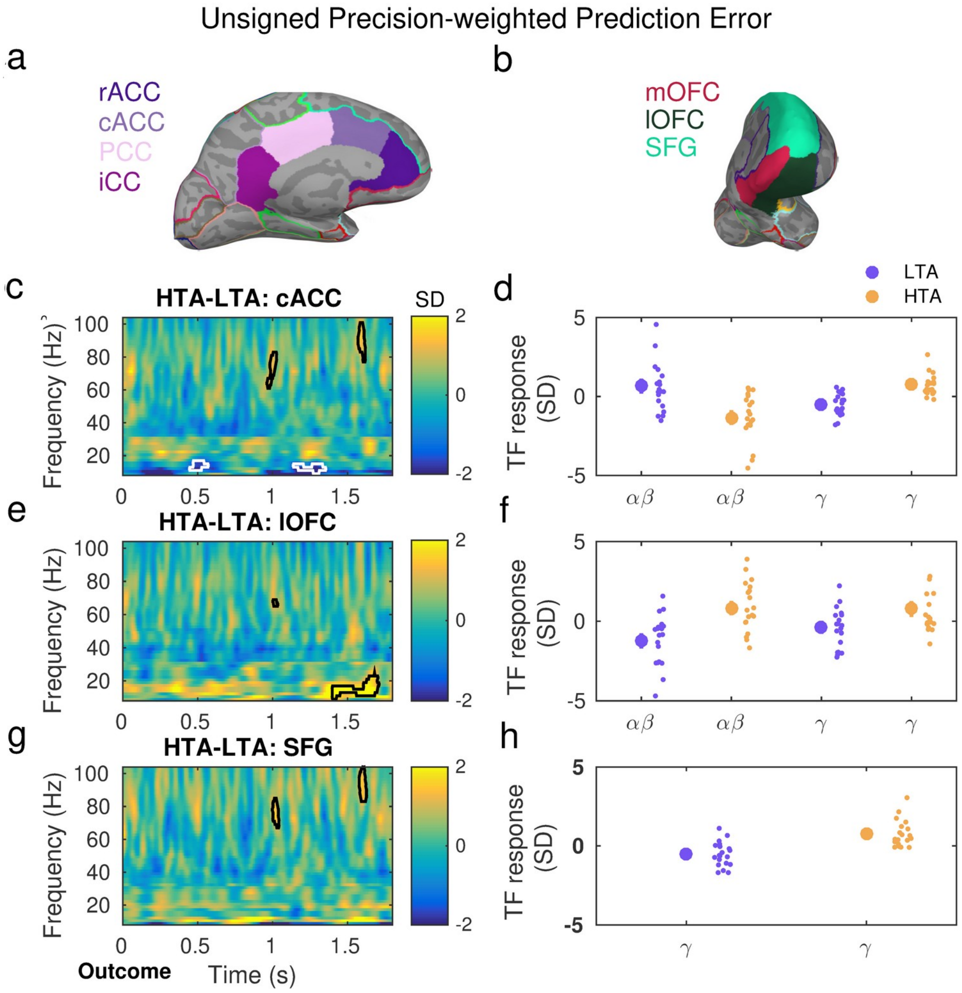

upplementary Figure 5. Same as **Figure 3**, but using a 40th-order Fourier basis set for the gamma-band convolution model. Between-group differences are denoted by the white and black contours (Significant clusters are FWER-controlled). All cluster effects extend for at least one cycle of the associated frequency.

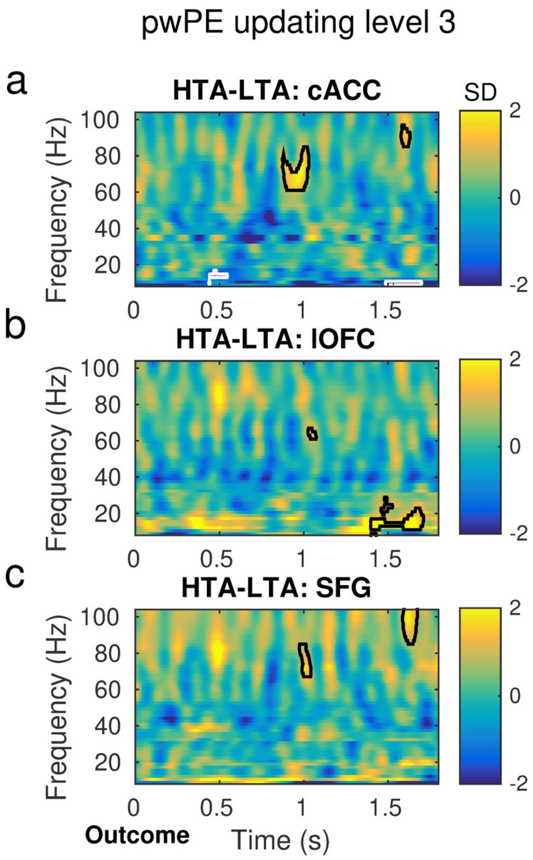

Supplementary Figure 6. Same as **Figure 3**, but including pwPE regressor ε_3 instead of $|\varepsilon_2|$ in the main convolution model. Between-group differences are denoted by the white and black contours (Significant clusters are FWER-controlled). All cluster effects extend for at least one cycle of the associated frequency.

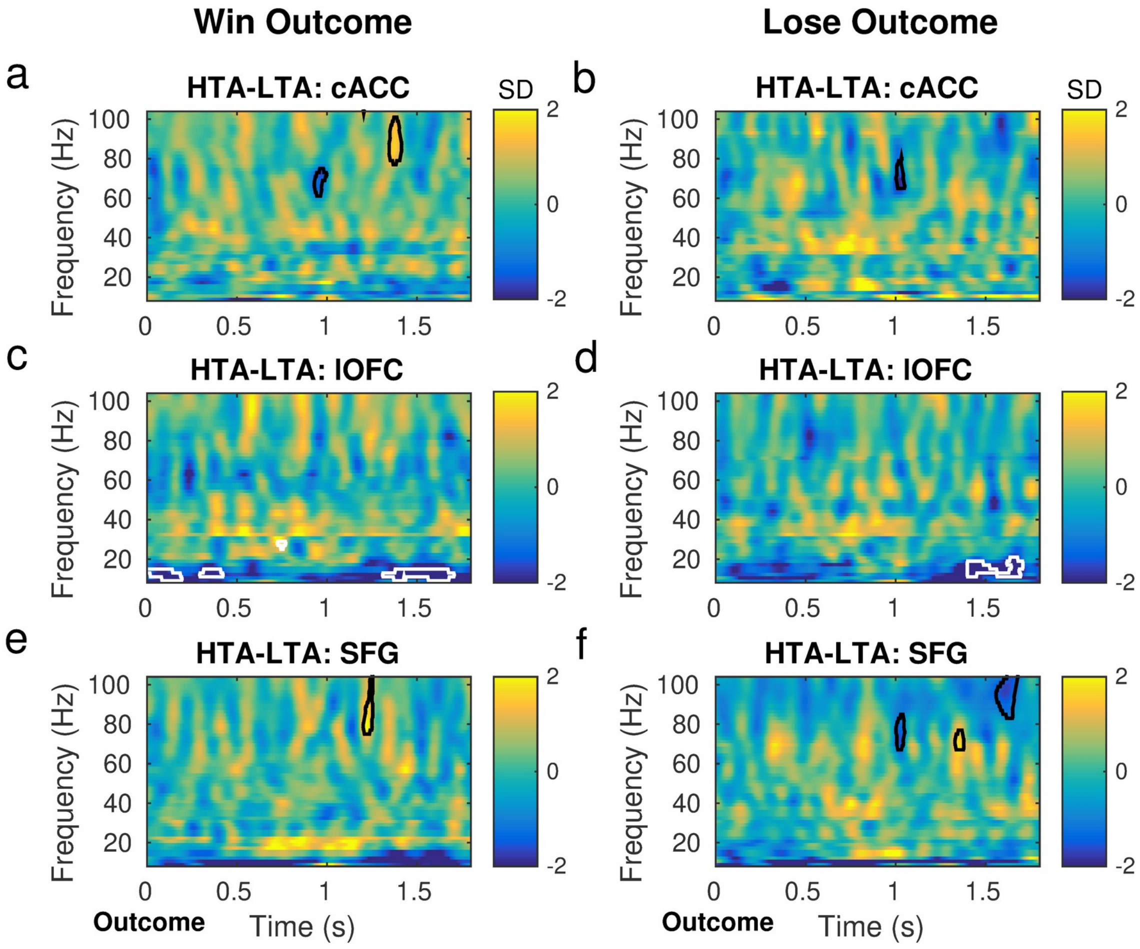

Supplementary Figure 7. Effect of win and lose regressors on gamma and alpha/beta power amplitude in pwPE convolution model. Panels **a, c, e** display the statistical results of the pwPE convolution model for the Win Outcome regressor. Between-group differences are denoted by the white and black contours (Significant clusters are FWER-controlled); Panels **b, d, f** same as **a, c, e** but for the Lose Outcome regressor. Relative to the unsigned pwPE effects, the polarity of most of the between-group differences for the win and lose outcome regressors was reversed: we observed a relative attenuation of gamma amplitude for HTA relative to LTA in the cACC and SFG, and an additional reduction in the beta frequency range in the lOFC.

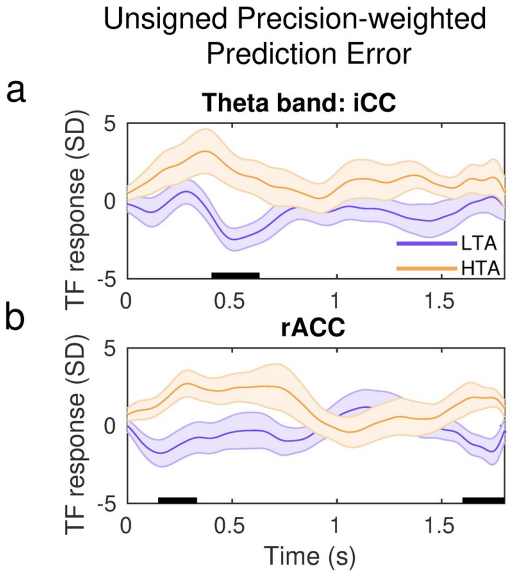

Suplementary Figure 8. Same as **Figure 3**, but running the convolution model in the theta (4–7 Hz) frequency range in an exploratory analysis. Between-group significant differences in TF responses to unsigned precision-weighted PEs (*P* = 0.034, *uncorrected*, rostral ACC and isthmus CC). HTA, high trait anxiety (yellow); LTA, low trait anxiety (purple).

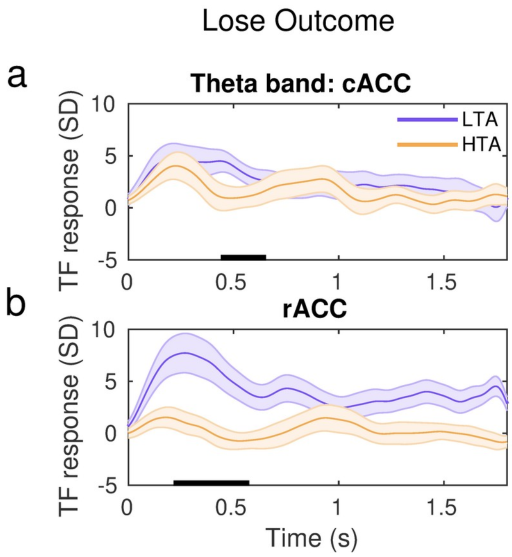

Supplementary Figure 9. Same as **Figure 3**, but running the convolution model in the theta (4–7 Hz) frequency range. Between-group significant differences in TF responses to the discrete Lose Outcome regressor (P = 0.041, *uncorrected*). HTA, high trait anxiety (yellow); LTA, low trait anxiety (purple).

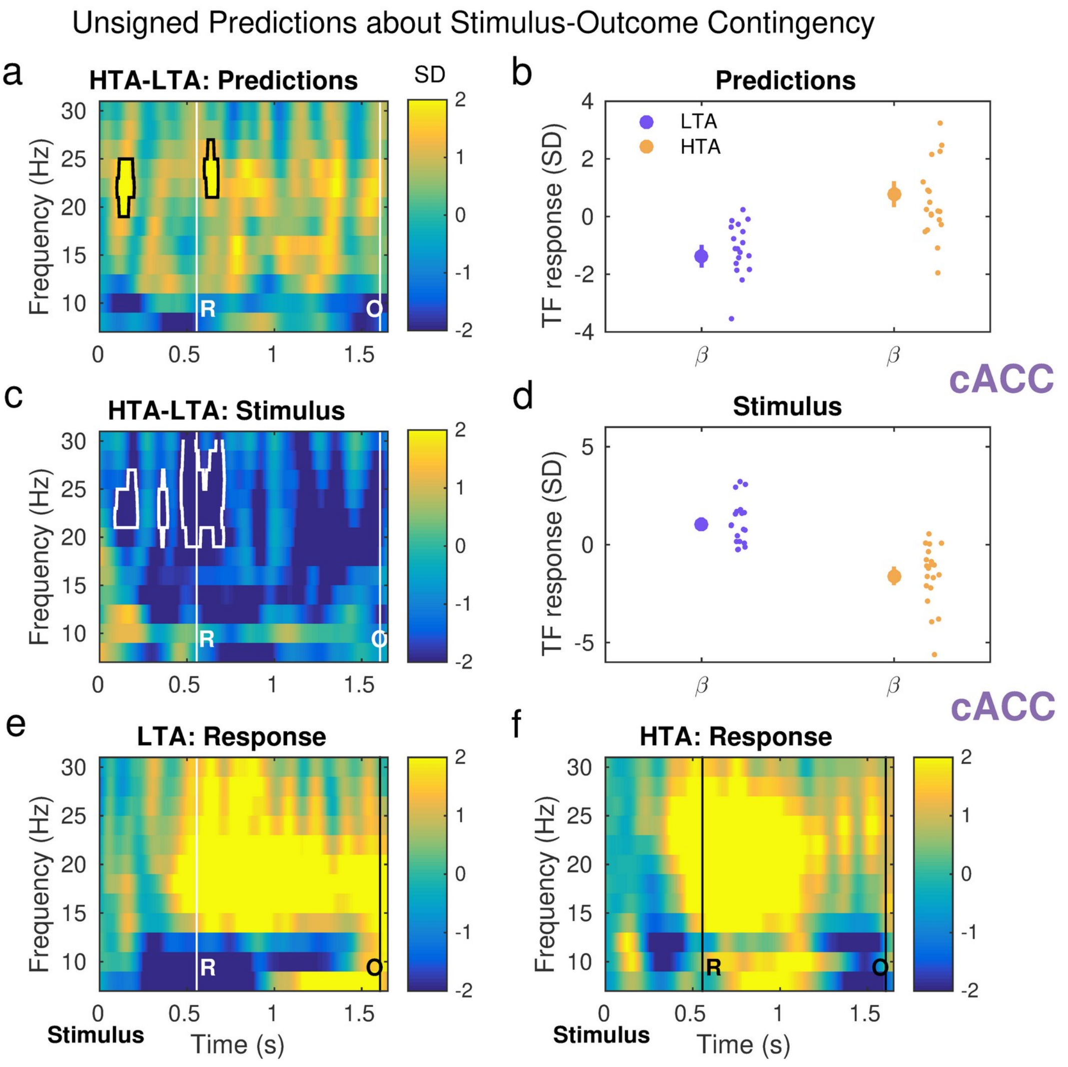

Supplementary Figure 10. Stimulus-locked beta activity reflects differential modulation with anxiety by predictions about the reward tendency. a) The regressor parametrising absolute predictions about the tendency towards a stimulus-reward contingency modulated beta activity differently in HTA and LTA participants (significant positive clusters at 100-200 ms and 600-680 ms post-stimulus, P = 0.005, FWER-controlled). This effect was limited to the right and left caudal ACC (right cACC results shown in the figure). The average response time in the combined sample was 555 (20) ms, which is denoted by a vertical line at the corresponding latency, labeled "R". The outcome was presented 1000 ms (± 200 ms) following the response, here denoted by the vertical line labeled "O". **b)** Average activity in the significant spectrotemporal clusters in a) shown separately for each group (LTA: purple; HTA: yellow). The large dot denotes mean and SEM as error bars. Individual dots represent individual participant average values. **c)** In the same convolution model, we observed that the discrete stimulus regressors induced a pronounced drop in beta activity in HTA when compared to LTA in the right cACC (shown here for blue stimulus on the left; similar results for blue stimulus on the right, not shown). This reduction was maintained throughout the post-stimulus interval (significant clusters from 100 until ~700 ms, before the feedback presentation; P = 0.001, FWER-controlled). **d)** Same as b) but for the stimulus (blue left) regressor. The relative reduction in beta activity shown in c) was associated with increases in the individual beta-band

TF responses to the stimulus regressor in LTA participants, and reductions in HTA participants. **e-f)** Stimulus-locked analysis of the response regressor (left response). LTA and HTA groups exhibited similar TF images in association with the response regressor. Panels E and F illustrate the expected alpha reduction prior to and during the response, followed by a pronounced beta rebound effect. There were no between-group differences in the TF images to the discrete regressors in any of our ROIs or additional anatomical labels (*P* > 0.05).

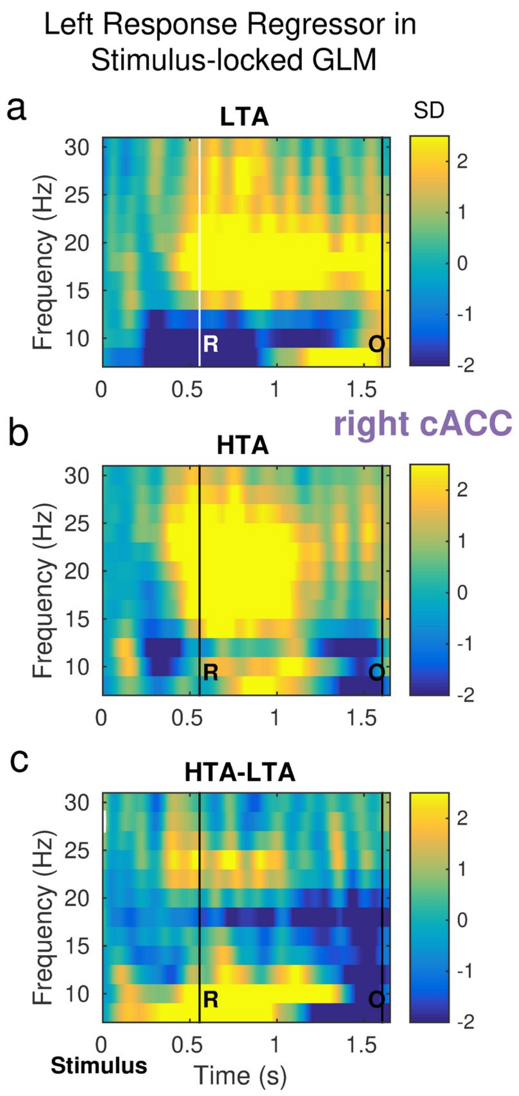

Supplementary Figure 11. Stimulus-locked analysis of the response regressor. a-b) Same as panels e and f in **Supplementary Figure 10. c)** No significant between-group differences in the TF images to the discrete regressors were observed in any of our ROIs or additional anatomical labels (*P* > 0.05).

Supplementary Tables

Table S1

Model	Prior	Mean	Variance
**HGF_3**	κ	1	0
	ω_2	−4	16
	ω_3	−7	16
	$\mu_2^{(0)}$	0	0
	$\sigma_2^{(0)}$	0.1	0
	$\mu_3^{(0)}$	1	0
	$\sigma_3^{(0)}$	1	0
	ζ	48	1
**HGF_2**	κ	0	0
	ω_2	−4	16
	ω_3	−7	0
	$\mu_2^{(0)}$	0	0
	$\sigma_2^{(0)}$	0.1	0
	$\mu_3^{(0)}$	1	0
	$\sigma_3^{(0)}$	1	0
	ζ	48	1
**$HGF\mu_3$**	κ	1	0
	ω_2	−4	16
	ω_3	−7	16
	$\mu_2^{(0)}$	0	0
	$\sigma_2^{(0)}$	0.1	0
	$\mu_3^{(0)}$	1	1
	$\sigma_3^{(0)}$	1	1

Table S1. Means and variances of the priors on perceptual parameters and starting values of the beliefs of the HGF models. Values are shown for 3-level HGF, 2-level HGF and $HGF_{\mu 3}$ models. Free parameters are estimated in their unbounded space. Parameters that are restricted to a confined interval are log-transformed, to allow for estimation in an unbounded space: ζ, $\sigma_2^{(0)}$, $\sigma_3^{(0)}$, κ. The prior variances are given in the space in which the parameters are typically estimated. We fixed some of these parameters, however (prior variance = 0 in the space in which they are estimated). As in recent work[14,84], the initial values of the belief trajectories were fixed in each individual for the 3-level HGF and 2-level HGF models: $\mu_2^{(0)}$, $\sigma_2^{(0)}$, $\mu_3^{(0)}$, $\sigma_3^{(0)}$. We estimated ω_2, ω_3 in each participant (ω_2 only for the 2-level HGF). The winning model $HGF_{\mu 3}$ had as free parameters ω_2, ω_3 , $\mu_3^{(0)}$, and $\sigma_3^{(0)}$, and the mapping from beliefs to decisions was a function of the inverse decision noise parameter $e^{-\mu_3^{k-1}}$.

Supplementary Results

Measures of anxiety

In our previous work, we assessed heart rate variability (HRV) as a proxy measure for state anxiety[14]. We calculated the coefficient of variation (CV = standard deviation/mean) of the difference intervals between consecutive R-peaks (inter-beat interval, IBI) extracted from the continuous ECG data as a metric of HRV. In previous empirical studies, states of anxiety have been shown to lower HRV and high-frequency HRV (HF-HRV, 0.15–0.4 Hz, see refs.[14,33,57,112]. However, experiments on the association between trait anxiety levels and HRV/HF-HRV metrics have reported both reductions[113-114] and small or inverse effects[115-116]. Here, we include a complimentary analysis of both HRV and HF-HRV to supplement our self-report measures of trait and state anxiety.

During preprocessing of the ECG signal, we extracted cardiac events (the QRS-complex, R wave peak) using the FieldTrip toolbox. Afterwards, we calculated the latency of each R-peak and the CV of the inter-beat interval (IBI), our proxy metric for HRV across both experimental task blocks. The CV for each participant was normalised to the resting state block (R1: baseline). To estimate the high-frequency content of the HRV (HF-HRV) we used the inter-beat-interval (IBI) time series. First, we interpolated at 1Hz with a spline function (order 3), and subsequently estimated spectral power using Welch's periodogram method (Hanning window, following ref.[117]). Estimates of power were normalised to the average power in R1 and converted to decibels (dB) for statistical analysis.

We conducted a 2 x 2 Group x Block (TB1, TB2) non-parametric factorial analysis of the HRV/HF-HRV measures. This analysis revealed a non-significant main effect of Group ($P = 0.57$), Block ($P = 0.21$) and interaction effect on HRV ($P = 0.91$, see **Supplementary Figure 2a**). Analysis of the spectral characteristics of the IBI time series also failed to obtain a significant main effect of Group ($P = 0.78$), Block ($P = 0.68$) and interaction effect ($P = 0.08$) on HF-HRV (**Supplementary Figure 2a**).

Subjective self-reported measures of state anxiety showed a significant main effect of the Group factor (HTA: mean 35.4, SEM 1.9; LTA: mean 27.9, SEM 1.1, $P = 0.0001$). There was no effect of the factor Block ($P = 0.23$) or interaction effect ($P = 0.22$). This analysis demonstrates that the HTA participants were subjectively more anxious than LTA during both task blocks. However, these subjective anxiety states did not result in statistical effects on physiological HRV/HF-HRV.

Total switch rate and lose-shift rate

Examining the total switch rate (trial-to-trial response switches of any kind[10]) as a proxy metric of exploratory response choices, several authors have reported impaired switching strategies in anxiety[10,13,43,60]. We therefore additionally assessed this measure and found that the overall switch

rate was significantly higher in HTA than LTA (P_{FDR} = 0.0134 < 0.05, Δ = 0.71, CI = [0.55, 0.82]; mean switch rate in each group and SEM: 0.24 [0.02] in HTA, 0.16 [0.02] in LTA). This result was, however, mainly accounted for by group differences in lose-shift rates, as shown in the main text.

A post-hoc analysis was conducted to explore whether the lose-shift rates or total switch rates could also be explained by a Group x Block interaction. This was not the case: no significant effect of Block or Interaction effect was found in either case ($P > 0.05$), but the significant Group effect on lose-shift and total switch rates remained (P_{FDR} = 0.0034 and 0.0120, respectively).

Bayesian Model Selection

While our BMS was conducted in the full sample of 39 participants, we confirmed that the $HGF_{\mu 3}$ model was equivalently the best model at describing responses independently in each group (LTA exceedance probability = 1; expected frequency = 0.91; HTA exceedance probability = 1; expected frequency = 0.89).

Parameter estimation in the HGF

We used simulations to evaluate the reliability of our estimates for the free model parameters in our implementation of the best fitting HGF model ($HGF_{\mu 3}$). In this model, the parameters that were estimated in each individual were ω_2, ω_3, $\mu_3^{(0)}$ and $\sigma_3^{(0)}$ (**Table S1**).

We simulated behavioural responses of 100 agents for six different values of ω_2 (total 600 simulations), and seven different values of ω_3 (total 700), when observing the input of one of our participants (ID zhanx_25). To determine the accuracy of the estimation of parameter $\mu_3^{(0)}$, we conducted similar simulations in 100 agents for six values of $\mu_3^{(0)}$.

These analyses were implemented using function tapas_simModel.m of the HGF toolbox (loop on om2 and om3, representing ω_2, ω_3, respectively; and loop on the number it iterations, N = 100):

```
sim = tapas_simModel(u, 'tapas_hgf_binary',  [NaN 0 1 NaN 1 1 NaN 0 0 1 1 NaN om2 om3],
'tapas_unitsq_sgm_mu3',123456789);
```

The simulated behavioural responses sim.y and the input u observed by participant #3 were then fitted with the tapas_fitModel.m function, similarly to the way we fitted the standard empirical data in our paritcipants:

```
est = tapas_fitModel(sim.y, u, hgf_binary_config, unitsq_sgm_mu3_config, optim_config)
```

with

```
optim_config = tapas_quasinewton_optim_config()
unitsq_sgm_mu3_config = tapas_unitsq_sgm_mu3_config()
hgf_binary_config = tapas_hgf_binary_config()
```

Note that in the above analyses the prior values of model parameters and initial values of the belief trajectories were modified in `tapas_hgf_binary_config.m` to correspond to the prior values for model HGF$_{\mu 3}$ given in **Table S1.**

This analysis demonstrated high accuracy for estimating ω_2 and $\mu_3^{(0)}$, while ω_3 was poorly recovered, as reported in previous work[14,99]. See figure below:

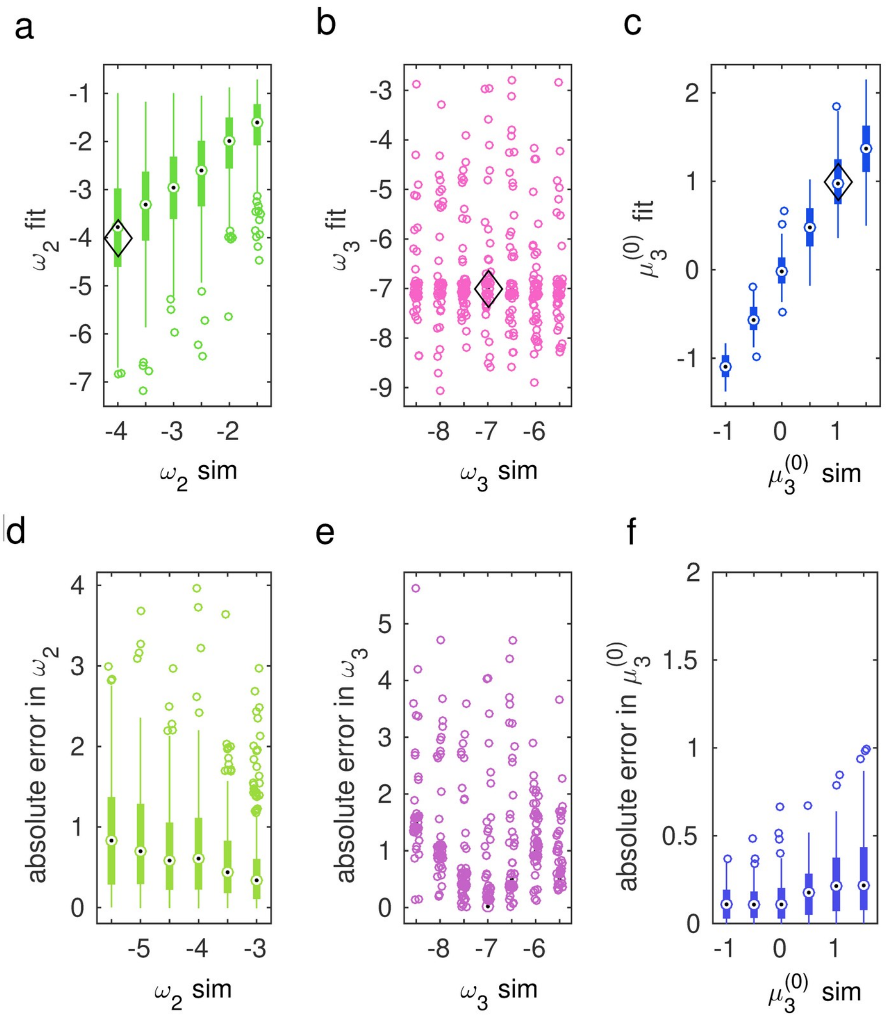

HGF parameter estimation using the input observed by one participant (ID zhanx_25). a-c) Boxplots (median, 25 and 75 percentiles) illustrating the results of parameter estimation for ω_2 (a), ω_3 (b) and $\mu_3^{(0)}$ (c). The x-axis represents the set parameters introduced in the simulated responses (labelled "sim"), while y-axis data reveal the corresponding estimated value of that same parameter (labelled "fit"). Parameters ω_2 and $\mu_3^{(0)}$ were estimated with high accuracy, as there was a high significant correlation between simulated and estimated (fit) values: Pearson R = 0.6306, $P \ll 1 \times 10^{-6}$ for ω_2, R = 0.9656, $P \ll 1 \times 10^{-6}$ for $\mu_3^{(0)}$. Parameter ω_3 was poorly estimated: R = -0.0242, P = 0.2668. The prior values of ω_2, ω_3 and $\mu_3^{(0)}$ used in the configuration file for estimating each parameter from the simulated responses were as defined in **Table**

S1 for the best fitting model (HGF$_{\mu3}$): ω_2 = -4, ω_3 = -7, respectively (variance 16 in both cases); $\mu_3^{(0)}$ = 1 (variance 1). Prior values are denoted by the diamond shape in the top panels.

A complementary analysis using simulated responses to observed inputs from a different participant (ID zhanx_3) provided similar results.

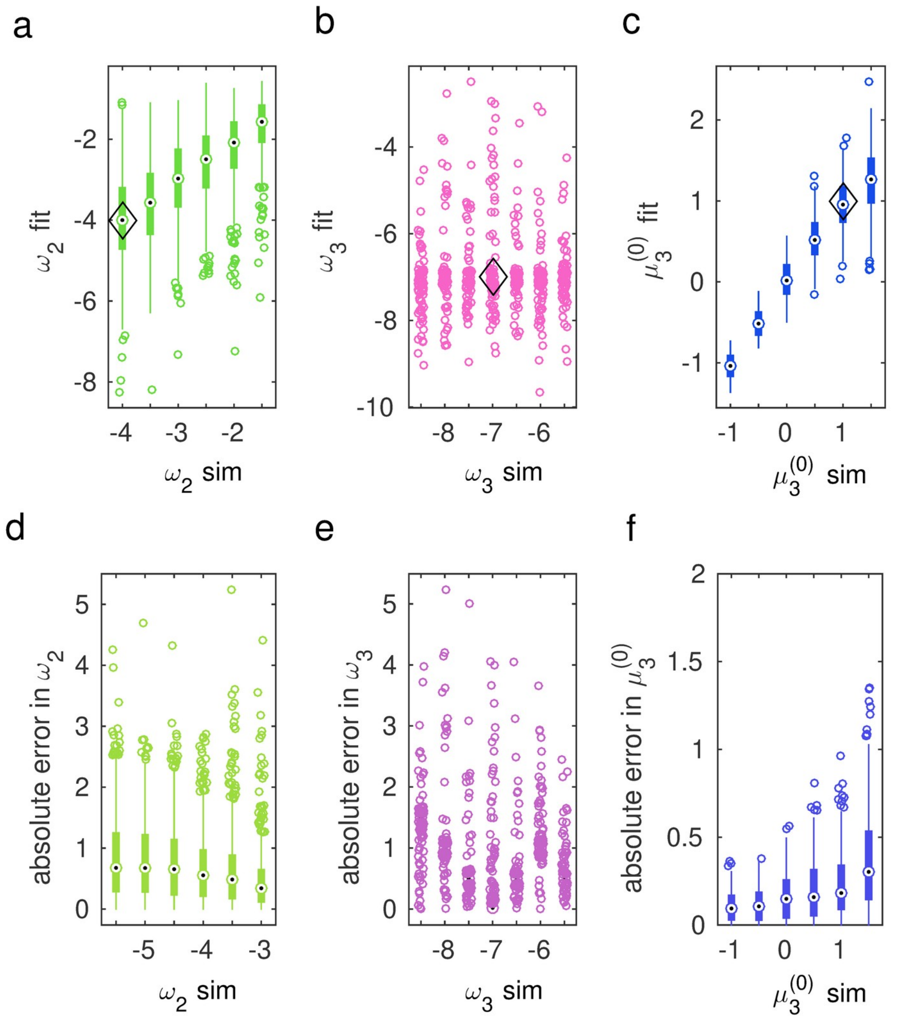

HGF parameter estimation using the input observed by one participant (ID zhanx_3). Same as the figure above. Estimation of parameters ω_2 and $\mu_3^{(0)}$ was highly accurate: Correlation between simulated and fitted value Pearson R = 0.6512, P << 1 x 10^{-6} for ω_2, R = 0.9468, P << 1 x 10^{-6} for $\mu_3^{(0)}$. Parameter ω_3 was also poorly estimated: R = 0.0019, P = 0.9019.

Validation analyses: Effect of pseudorandomised order of contingency mappings

Each participant observed a different combination of five phases of stimulus-outcome probability mappings in each block. The possible mappings in each block were 0.9/0.1, 0.7/0.3, 0.5/0.5,

0.3/0.7, 0.1/0.9, reflecting the probability that blue/orange images are rewarding. Our task script did pseudorandomly generate the order of these phases independently in each block and participant. Accordingly, it was possible that the same probability mapping would occur at the end of block 1 and, after a break, at the beginning of block 2.
Visual inspection of **Supplementary Figure 1a** reveals that three HTA participants and four LTA participants did not experience a change in contingency mapping from block 1 to block 2. On the other hand, 16/19 HTA and 16/20 LTA participants experienced a contingency mapping change from block 1 to block 2.

To exclude the possibility that group differences in the pseudorandomised order of contingency mappings could explain the behavioural and computational results, we conducted a series of validation analyses. First, we assessed whether there were between-group differences in the true experienced volatility, as three HTA participants and four LTA participants did not experience a change in contingency mapping from block 1 to block 2, which slightly decreased their overall true volatility relative to the remaining 16 HTA and 16 LTA participants.

We evaluated between-group differences in the experienced true volatility by computing Bayes Factors (BF) using the bayesFactor toolbox (https://github.com/klabhub/bayesFactor) in MATLAB. This toolbox implements tests that are based on multivariate generalisations of Cauchy priors on standardised effects[118].
For between-group comparisons of a dependent variable (DV), we calculated the BF on the model DV ~ 1 + group, where DV is explained by a fixed effect of group (HTA, LTA). The model was fitted using the fitlme function of the MATLAB Statistics toolbox. Computing BF allowed us to quantify the evidence in support of the alternative hypothesis (full model, in our case assessing the main effect of the group) relative to the null model (intercept-only model, i.e., DV ~ 1).
This approach was implemented using measures of the true experienced volatility as DV.

BF values were interpreted as in ref.[119]. As BF is the ratio between the probability of the data being observed under the alternative hypothesis and the probability of the same data under the null hypothesis,

$$BF_{10} = \text{likelihood of data given H1} / \text{likelihood of data given H0}$$

a BF_{10} of 20 would indicate strong evidence for the alternative hypothesis. On the other hand, BF of 0.05 would provide strong evidence for the null hypothesis (see Table 1 by ref.[119] for further details).

We estimated the following quantities to reflect the true experienced volatility by each participant:

(a) **Number of switches in contingency mapping**: this was 9 in all participants except for 3 HTA and 4 LTA participants, who observed 8 switches respectively. We found moderate evidence for the null hypothesis, supporting that this quantity is equal in both groups: BF_{10} = 0.21 (Bayes factor in range 1/3 – 1/10 is associated with moderate evidence for H0) . The p-value obtained using between-group permutation tests is *P* = 0.6943. Descriptive statistics indicated that HTA participants had 8.84 (SEM 0.09) switches over 320 trials, while LTA participants had 8.80 (SEM 0.09)

(b) **Average number of trials after which participants experienced a change in the contingency mapping**: 32.80 (0.42) for HTA, 32.76 (0.49) for LTA. We obtained moderate evidence in support of the null hypothesis, BF_{10} = 0.2057 (*P* = 0.9670).

Accordingly, there was moderate evidence in favour of the null hypothesis that both anxiety groups experienced an equal amount of true volatility, despite the contingency mappings having a pseudorandomised order in each participant and block.

Second, the group-average contingency mapping is displayed in **Supplementary Figure 1b**. We observe that the group-average Pr(win|blue) trajectory closely overlaps in both groups throughout the task, except during trials 90-95. To assess whether the probabilistic relationships were different in each group, we conducted a second Bayes factor analysis.

We estimated BF for a factorial analysis to assess the effect of group and trial bins on Pr(win|blue). To this aim, we used as full model DV ~ 1 + group*bin, which includes three categorical fixed effects: group, bin and interaction group:bin. Next, we constructed the restricted models by excluding each of the main or interaction effects. We then computed the ratio of the full model and each restricted model. The resulting BF provided evidence for either main effect (group, bin) or interaction effect.

Here, we transformed the 320 trials into a categorical variable: 10 bins of 32 trials. Thus, we assessed the BF of a 10 x 2 ANOVA model with factors bin (10 levels: average within each bin of 32 trials) and group (HTA, LTA). The results revealed a BF=6.7482e-04 for the main effect of bin, demonstrating extreme evidence for H0 and supporting that the average Pw(blue|win) was not modulated across bins.

The main effect of group was associated with a BF = 0.0655, providing strong evidence in favour of H0 that the population mean in both groups was equal.

Last, we obtained a BF = 0.0015 for the interaction effect. This demonstrated extreme evidence for a lack of interaction effect.

Our BF analyses above demonstrate that, although we pseudorandomised the contingency phases separately in each individual and block, and 3/19 HTA and 4/20 LTA participants did not experience

a switch from block 1 to 2, this did not contribute to group differences in true experienced volatility or the stimulus-outcome probabilistic relationships.

We complemented the BF analyses with a reanalysis of the main behavioural and computational variables in the two subsamples of 16 HTA and 16 LTA participants who experienced exactly nine switches in contingency mappings, thus excluding the 3 HTA and 4 LTA participants who observed a repetition of the contingency mapping from block 1 to block 2. This analysis effectively regresses out any potential differences in the experienced true contingency.

Reanalysis of computational variables in **Figure 2**:

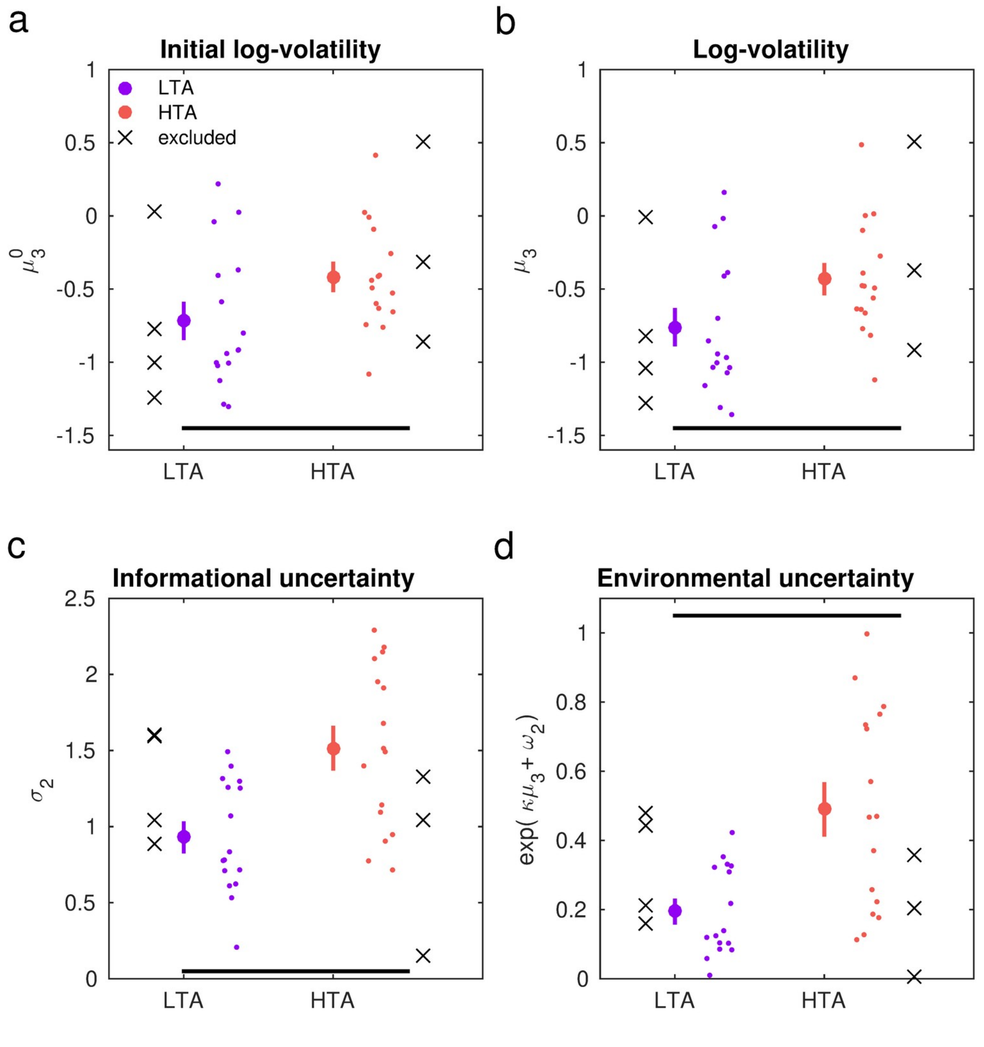

The figure above is similar to **Figure 2e-h** but represents the group analysis with the subsamples of 16 HTA and 16 LTA participants observing 9 switches in probabilistic mapping. The participants who observed 8 switches are marked as “excluded” and denoted by the crosses. Visual inspection of the excluded participants in panels A and B indicates that some participants had a large

expectation on log-volatility despite being exposed to slightly smaller true volatility (8 switches instead of 9).

Between-group statistical analysis in the 16-16 subsamples demonstrated that:

(A) HTA individuals (red) had a greater initial expectation or prior on log-volatility than LTA (purple, $P_{FDR} = 0.0454 < 0.05$, $\Delta = 0.70$, CI = [0.52, 0.87]; group effects denoted by the black line at the bottom).

(B) Over time, the posterior mean on log-volatility (μ_3) in HTA remained significantly higher relative to LTA ($P_{FDR} = 0.0378 < 0.05$, $\Delta = 0.72$, CI = [0.52, 0.88]).

(C) Informational (estimation) belief uncertainty about the stimulus outcome tendency was greater in HTA compared with LTA ($P_{FDR} = 0.0012 < 0.05$, $\Delta = 0.81$, CI = [0.62, 0.92]).

(E) The HTA individuals were also significantly more uncertain about the environment ($P_{FDR} = 0.0008 < 0.05$, $\Delta = 0.82$, CI = [0.65, 0.92]).

Using the HTA and LTA subsamples, we also replicated the result that HTA had a smaller win rate in the first block ($P = 0.0370$, non-parametric effect size $\Delta = 0.7031$) but not in the second one ($P = 0.9181$, $\Delta = 0.5039$), when compared to LTA.

Accordingly, when excluding participants with 8 instead of 9 contingency mapping changes, we replicate the main behavioural and computational results of the study (note however the smaller sample sizes). Crucially, HTA participants in the total sample and subsample had a greater expectation on log-volatility, primarily due to an initially higher estimate. Thus, the switch in contingencies from block 1 to block 2 has negligible effects on the inferred volatility estimate in the task. It is also important to note that HTA individuals in the total sample and subsample had a greater expectation on informational belief uncertainty (σ_2). Accordingly, they update their beliefs about the tendency of the stimulus-outcome contingencies faster, using greater steps. This computational result is the main driver of the neural oscillatory effects on pwPE, which we also replicate in the subsamples:

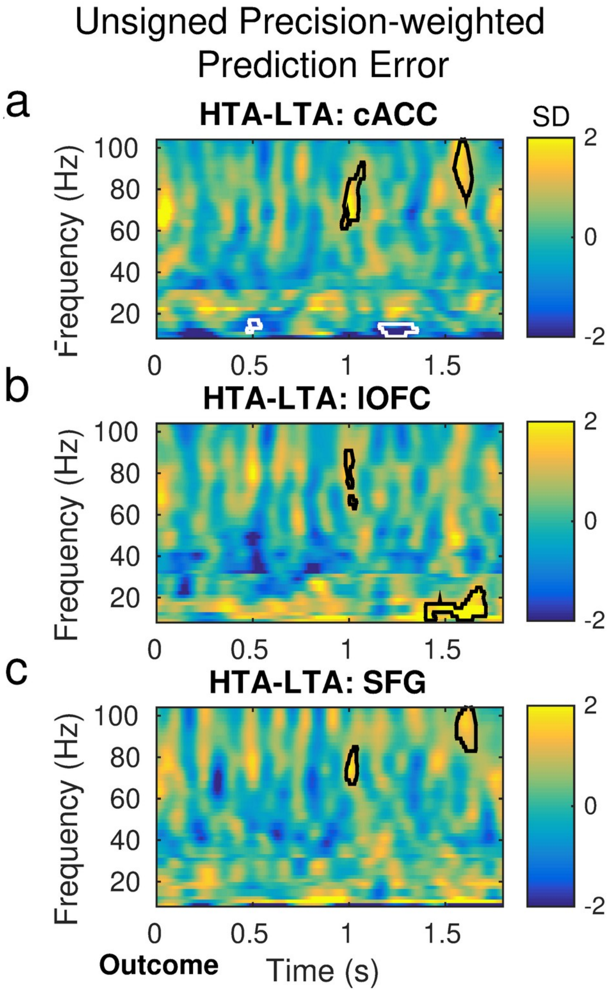

This figure is similar to **Figure3**, panels c, e, g but using the 16 HTA and 16 LTA subsamples. The black and white contours denote between-group effects with cluster-based permutation testing, FWER-controlled.

Supplementary References

112. Thayer, J. F., Friedman, B. H., & Borkovec, T. D. Autonomic characteristics of generalized anxiety disorder and worry. *Biol Psychiatry.* **39**(4), 255-266 (1996).

113. Miu, A. C., Heilman, R. M., & Miclea, M. Reduced heart rate variability and vagal tone in anxiety: trait versus state, and the effects of autogenic training. *Auton Neurosci*. **145**(1-2), 99-103 (2009).

114. Mujica-Parodi, L. R. *et al*. Limbic dysregulation is associated with lowered heart rate variability and increased trait anxiety in healthy adults. *Hum Brain Mapp.* **30**(1), 47-58 (2009).

115. Dishman, R. K., *et al*. Heart rate variability, trait anxiety, and perceived stress among physically fit men and women. *Int J Psychophysiol.* **37**(2), 121-133 (2000).

116. Narita, K., *et al*. Interactions among higher trait anxiety, sympathetic activity, and endothelial function in the elderly. *J Psychiatr Res.* **41**(5), 418-427 (2007).

117. Rebollo, I., Devauchelle, A. D., Béranger, B., & Tallon-Baudry, C. Stomach-brain synchrony reveals a novel, delayed-connectivity resting-state network in humans. *Elife.* **7**, e33321 (2018).

118. Rouder, J. N., Morey, R. D., Speckman, P. L., & Province, J. M. Default Bayes factors for ANOVA designs. *J Math Psychol.* **56**(5), 356-374 (2012).

119. Andraszewicz, S., *et al.* (2015). An introduction to Bayesian hypothesis testing for management research. *J Managment.* **41**(2), 521-543.
