# Supplementary material for: Anterior cingulate and medial prefrontal cortex oscillations underlie learning alterations in trait anxiety in humans
Source: Commun Biol. 2023 Mar 15;6:271. doi: 10.1038/s42003-023-04628-1 (PMC10017780; doi:10.1038/s42003-023-04628-1)
Supplement: Supplementary file 2 — Reporting Summary [file 42003_2023_4628_MOESM2_ESM.pdf]

nature portfolio

Corresponding author(s): Maria Herrojo Ruiz

Last updated by author(s): Feb 13, 2023

Reporting Summary

Nature Portfolio wishes to improve the reproducibility of the work that we publish. This form provides structure for consistency and transparency in reporting. For further information on Nature Portfolio policies, see our Editorial Policies and the Editorial Policy Checklist.

Statistics

For all statistical analyses, confirm that the following items are present in the figure legend, table legend, main text, or Methods section.

n/a	Confirmed	
☐	☒	The exact sample size (*n*) for each experimental group/condition, given as a discrete number and unit of measurement
☐	☒	A statement on whether measurements were taken from distinct samples or whether the same sample was measured repeatedly
☐	☒	The statistical test(s) used AND whether they are one- or two-sided<br>*Only common tests should be described solely by name; describe more complex techniques in the Methods section.*
☐	☒	A description of all covariates tested
☐	☒	A description of any assumptions or corrections, such as tests of normality and adjustment for multiple comparisons
☐	☒	A full description of the statistical parameters including central tendency (e.g. means) or other basic estimates (e.g. regression coefficient) AND variation (e.g. standard deviation) or associated estimates of uncertainty (e.g. confidence intervals)
☐	☒	For null hypothesis testing, the test statistic (e.g. F, t, r) with confidence intervals, effect sizes, degrees of freedom and P value noted<br>*Give P values as exact values whenever suitable.*
☐	☒	For Bayesian analysis, information on the choice of priors and Markov chain Monte Carlo settings
☒	☐	For hierarchical and complex designs, identification of the appropriate level for tests and full reporting of outcomes
☐	☒	Estimates of effect sizes (e.g. Cohen's *d*, Pearson's *r*), indicating how they were calculated

Our web collection on statistics for biologists contains articles on many of the points above.

Software and code

Policy information about availability of computer code

Data collection	Data were collected using Matlab 2020b, Psychtoolbox-3.0.16 and Elekta software (MaxfilterTM)
Data analysis	This uses Freesurfer 6.0, MNE-Python version 1.2.2, SPM 12, FieldTrip toolbox 2021, TAPAS Version 6.0.1, and custom-made code based on these software packages and deposited in the Open Science Framework Data Repository under the accession code wsjgk.

For manuscripts utilizing custom algorithms or software that are central to the research but not yet described in published literature, software must be made available to editors and reviewers. We strongly encourage code deposition in a community repository (e.g. GitHub). See the Nature Portfolio guidelines for submitting code & software for further information.

Data

Policy information about availability of data

All manuscripts must include a data availability statement. This statement should provide the following information, where applicable:

- Accession codes, unique identifiers, or web links for publicly available datasets
- A description of any restrictions on data availability
- For clinical datasets or third party data, please ensure that the statement adheres to our policy

Behavioural and computational modelling data are deposited in the Open Science Framework Data Repository under the accession code wsjgk.

Human research participants

Policy information about studies involving human research participants and Sex and Gender in Research.

Reporting on sex and gender	We collected information on biological sex as self-reported by the participants. We balanced the proportion of males and females in each experimental and control group. We report in the manuscript that: "the experimental groups were balanced in terms of age and sex. The high trait anxiety group (HTA, mean age 22.6, SEM = 1.1) consisted of 12 females, while the low trait anxiety group (LTA, mean age 23.7, SEM = 1.0) consisted of 12 females. " We have not analysed specific sex effects on our dependent variables.
Population characteristics	We collected information on age and balanced the age range in each experimental and control group. See above.
Recruitment	Through adds posted on the university campus and on social media (2021)
Ethics oversight	Higher School of Economics (2020-2021)

Note that full information on the approval of the study protocol must also be provided in the manuscript.

Field-specific reporting

Please select the one below that is the best fit for your research. If you are not sure, read the appropriate sections before making your selection.

☒ Life sciences ☐ Behavioural & social sciences ☐ Ecological, evolutionary & environmental sciences

For a reference copy of the document with all sections, see nature.com/documents/nr-reporting-summary-flat.pdf

Life sciences study design

All studies must disclose on these points even when the disclosure is negative.

Sample size	Our sample size was estimated using the behavioural and EEG data from our recent work on decision making in state anxiety (Hein et al., 2020, 2021). MATLAB function sampsizepwr (two-tailed t-test) was used to estimate the minimum sample size for a statistical power of 0.80, with an a of 0.05. This function was evaluated on the HGF model parameter ω2 (the low-level tonic log-volatility estimate) and the beta activity modulation to pwPE, resulting in a minimum of 16 participants in each group (high, low anxiety). In the current MEG study, we recruited 20 and 19 participants in the LTA and HTA groups, respectively.
Data exclusions	No data exclusion.
Replication	No replication study has been conducted yet using the same design. However, we've conducted a separate study with a similar decision-making task in trait anxiety in the UK in 2022 (pairing decision-making to motor learning) and we've replicated the Bayesian model selection results (manuscript in preparation).
Randomization	Participants were pseudorandomly allocated to the experimental and control groups, while the biological sex and age were balanced between groups.
Blinding	There was no experimental/control treatment and therefore blinding was not implemented. Participants were pre-screened into each group based on their anxiety scores.

Reporting for specific materials, systems and methods

We require information from authors about some types of materials, experimental systems and methods used in many studies. Here, indicate whether each material, system or method listed is relevant to your study. If you are not sure if a list item applies to your research, read the appropriate section before selecting a response.

Materials & experimental systems

n/a	Involved in the study	
☒	☐	Antibodies
☒	☐	Eukaryotic cell lines
☒	☐	Palaeontology and archaeology
☒	☐	Animals and other organisms
☒	☐	Clinical data
☒	☐	Dual use research of concern

Methods

n/a	Involved in the study	
☒	☐	ChIP-seq
☒	☐	Flow cytometry
☒	☐	MRI-based neuroimaging
